# Monomeric eNAMPT in the development of experimental diabetes in mice: a potential target for type 2 diabetes treatment

**DOI:** 10.1007/s00125-016-4076-3

**Published:** 2016-08-19

**Authors:** Julius Kieswich, Sophie R. Sayers, Marta F. Silvestre, Steven M. Harwood, Muhammad M. Yaqoob, Paul W. Caton

**Affiliations:** 1 Translational Medicine and Therapeutics, William Harvey Research Institute, Bart’s and the London School of Medicine and Dentistry, Queen Mary University of London, London, UK; 2 Diabetes Research Group, Division of Diabetes and Nutritional Sciences, King’s College London, Hodgkin Building, Guy’s Campus, London, SE1 1UL UK; 3 Faculdade de Medicina da Universidade de Lisboa, Lisbon, Portugal; 4 Human Nutrition Unit, University of Auckland, Auckland, New Zealand

**Keywords:** Beta cell, Extracellular nicotinamide phosphoribosyltransferase, eNAMPT, Inflammation, Islet, Type 2 diabetes

## Abstract

**Aims/hypothesis:**

Serum extracellular nicotinamide phosphoribosyltransferase (eNAMPT) concentrations are elevated in type 2 diabetes. However, the relationship between abnormally elevated serum eNAMPT and type 2 diabetes pathophysiology is unclear. eNAMPT circulates in functionally and structurally distinct monomeric and dimeric forms. Dimeric eNAMPT promotes NAD biosynthesis. The role of eNAMPT-monomer is unclear but it may have NAD-independent proinflammatory effects. However, studies of eNAMPT in type 2 diabetes have not distinguished between monomeric and dimeric forms. Since type 2 diabetes is characterised by chronic inflammation, we hypothesised a selective NAD-independent role for eNAMPT-monomer in type 2 diabetes.

**Methods:**

Two mouse models were used to examine the role of eNAMPT-monomer in type 2 diabetes; (1) a mouse model of diabetes fed a high-fat diet (HFD) for 10 weeks received i.p. injections with an anti-monomeric-eNAMPT antibody; and (2) lean non-diabetic mice received i.p. injections with recombinant monomeric eNAMPT daily for 14 days.

**Results:**

Serum monomeric eNAMPT levels were elevated in HFD-fed mouse models of diabetes, whilst eNAMPT-dimer levels were unchanged. eNAMPT-monomer neutralisation in HFD-fed mice resulted in lower blood glucose levels, amelioration of impaired glucose tolerance (IGT) and whole-body insulin resistance, improved pancreatic islet function, and reduced inflammation. These effects were maintained for at least 3 weeks post-treatment. eNAMPT-monomer administration induced a diabetic phenotype in mice, characterised by elevated blood glucose, IGT, impaired pancreatic insulin secretion and the presence of systemic and tissue inflammation, without changes in NAD levels.

**Conclusions/interpretation:**

We demonstrate that elevation of monomeric-eNAMPT plays an important role in the pathogenesis of diet-induced diabetes via proinflammatory mechanisms. These data provide proof-of-concept evidence that the eNAMPT-monomer represents a potential therapeutic target for type 2 diabetes.

**Electronic supplementary material:**

The online version of this article (doi:10.1007/s00125-016-4076-3) contains peer-reviewed but unedited supplementary material, which is available to authorised users.

## Introduction

Type 2 diabetes is characterised by the presence of peripheral insulin resistance and pancreatic beta cell dysfunction [1]. Determining the precise pathophysiological mechanisms responsible for these processes is essential for the development of novel therapeutics.

Serum concentrations of extracellular nicotinamide phosphoribosyltransferase (eNAMPT; also referred to as visfatin/pre-B cell colony-enhancing factor [PBEF]) are commonly elevated in type 2 diabetes patients [2], whilst raised eNAMPT levels strongly correlate with declining beta cell function [3]. Therefore, a pathophysiological role is implied for eNAMPT in type 2 diabetes. However, other studies have reported both insulin sensitising and beta cell protective effects of eNAMPT [4–7]. Therefore, the precise relationship between elevated eNAMPT and type 2 diabetes remains unresolved.

Nicotinamide phosphoribosyltransferase exists in intracellular (iNAMPT) and extracellular (eNAMPT) forms. iNAMPT is widely expressed and is a well-characterised NAD biosynthetic enzyme [8]. In contrast, the function of eNAMPT is unclear, although putative proinflammatory [9, 10], insulin-mimetic [11] and NAD biosynthetic functions [5, 12, 13] have been described. These disparate putative functions are controversial and have been challenged [11, 14], but may be explained by the presence of structurally and functionally distinct monomeric (50 kDa) and dimeric (100 kDa) forms of eNAMPT. Dimerisation is reportedly essential for the biosynthetic functions of NAMPT [5, 15]. The eNAMPT-monomer has potential NAD-independent proinflammatory effects. However, the precise structure–function relationships have not yet been fully investigated, particularly within the context of type 2 diabetes pathophysiology.

Given the crucial role of chronic inflammation in type 2 diabetes pathophysiology, we hypothesised that eNAMPT-monomer levels will be selectively elevated in type 2 diabetes and by potentially acting in a proinflammatory manner, may play a key role in type 2 diabetes pathophysiology.

## Methods

### Animal studies

For immunoneutralisation experiments, 8-week-old male C57Bl/6 mice (Charles River, Margate, UK) were fed a high-fat diet (HFD; 60% wt/wt fat; 58Y1; Test Diets, St. Louis, MO, USA) or a control diet (CON) for 10 or 13 weeks, and then injected i.p. with a rabbit polyclonal mouse anti-eNAMPT antibody (2.5 μg/ml; LS-C48964; LifespanBio, WA, USA) or a non-immune IgG equivalent (four separate doses during weeks 9–10). Antibodies were validated via immunoprecipitation and immunoblotting. Mice were then killed either directly post-treatment (at 10 weeks) or 3 weeks later (at 13 weeks; *n* = 24/group)

For experimental elevation of eNAMPT and nicotinamide mononucleotide (NMN), 8-week old male C57Bl/6 mice were i.p. injected daily with either recombinant eNAMPT (5 ng/ml; Adipogen, Seoul, South Korea), NMN (500 mg/kg body weight; Sigma, Poole, UK) or the equivalent volume of NaCl 154 mmol/l (Sigma) for 14 days (*n* = 8/group).

Mice were maintained on a 12 h light/12 h dark cycle. Animal experiments were conducted in accordance with UK Home Office Animals (Scientific Procedures) Act 1986, with local ethical committee approval. Experimenters were not blinded to group assignment or outcome assessment. Specific randomisation of animals into groups was not carried out. No data, samples or animals were excluded from the study.

### IPGTT and in vivo insulin secretion

Mice were fasted overnight then injected i.p. with 2 g/kg body weight 25% wt/vol. dextrose (Sigma). Glucose was measured in tail vein blood samples (Accuchek, Roche Diagnostics, Burgess Hill, UK) between 0 and 120 min. Additional blood was collected during the IPGTT to estimate the insulin secretory response to glucose.

### Model assessments of insulin resistance

Whole-body insulin resistance and sensitivity were assessed using three equations: (1) insulin resistance (IR) = fasting glucose (mmol/l) × fasting insulin (pmol/l); (2) HOMA-IR (log HOMA-IR = log_10_ [fasting insulin (pmol/l) × fasting glucose (mmol/l) ÷ 22.5]); and (3) QUICKI = (1/log_10_ [fasting insulin (pmol/l)] + log_10_ [fasting glucose(mmol/l)]).

### Blood chemistry

Blood was obtained by cardiac puncture. Serum and liver triacylglycerol levels were determined using a colourimetric assay kit (Cayman Chemical, Ann Arbor, MI, USA). Serum NEFA levels were measured using a fluorometric assay (Abcam, Cambridge, UK). ELISA kits were used to measure serum insulin (Mercodia, Uppsala, Sweden), eNAMPT (Caltag, Buckingham, UK), and TNF-α, IL-1β and MCP1 (eBioscience, Hatfield, UK).

### Serum NMN and tissue NAD levels

Serum NMN was detected fluorometrically by HPLC, using a modified version of a previously described methodology (see electronic supplementary material [ESM] Methods) [16]. Tissue NAD levels were determined using a NAD/NADH quantification kit (Sigma).

### Immunoblotting

Immunoblotting was conducted as previously described [17] using primary antibodies against NAMPT (Sigma or Bethyl Laboratories, Montgomery, TX, USA), p-Akt(Ser^473^) and total Akt (both Cell Signaling Technologies, Danvers, MA, USA). All primary antibodies were rabbit anti-mouse polyclonal antibodies and were used at a 1:1000 dilution. Validation was conducted via immunoblot. NAMPT immunoprecipitation was carried out using a Catch and Release Reversible Immunoprecipitation System (Millipore, Watford, UK).

### Quantitative RT-PCR

Gene expression was determined by TaqMan or SYBR green quantitative RT-PCR (qRT-PCR) [17], using ΔΔCt methodology, and normalised against *18S* ribosomal RNA levels (Applied Biosystems, Warrington, UK). Changes in gene expression are normalised to control. For details of primers (Eurogentec, Southampton, UK), see ESM Table 1

### Mouse islet isolation and insulin secretion

Mouse pancreases were digested in 2 ml Hanks Buffered Salt Solution (HBSS) containing 1 mg/ml collagenase P and 0.15 mg/ml DNAse I (both Roche Diagnostics). Islets were hand-picked and transferred into RPMI 1640 medium for RNA extraction or insulin secretion assays. For islet insulin secretion assays, batches of eight size-matched islets were pre-incubated for 1 h at 37°C in HBSS containing 3 mmol/l glucose, 10 mmol/l HEPES (pH 7.4) and 0.2% BSA (wt/vol.). For glucose-stimulated insulin secretion (GSIS) analysis, islets were incubated for 1 h at 37°C in HBSS containing 10 mmol/l HEPES (pH 7.4) and 0.2% BSA, supplemented with 3 mmol/l or 17 mmol/l glucose. After 1 h, the medium was collected for determination of insulin levels by ELISA (see ESM Methods ‘islet isolation’ and ‘insulin secretion ex vivo’ for further details).

### Immunofluorescence analysis of mouse pancreatic sections

Immunostaining was conducted as previously described [7]. Briefly, the whole pancreas was fixed in buffered formalin, embedded in paraffin, cut into sections and stained with guinea pig anti-insulin (1:100 dilution; Abcam) and/or rabbit anti-p-P38 (1:1600 dilution; Cell Signaling Technologies) antibodies. Sections were then mounted on glass cover slips and analysed using a Leica DM5000 epifluorescence microscope with Leica Application Suite software (Leica, Milton Keynes, UK) (see ESM Methods).

### MIN6 cell culture and treatment

MIN6 beta cells were incubated with 2–10 ng/ml eNAMPT (Adipogen) with or without anti-eNAMPT antibody (2.5 μg/ml; LS-C48964) and analysed for changes in insulin secretion or NAD levels (see ESM Methods). MIN6 cells were free from mycoplasma contamination

### Isolation of white adipocytes and the stromal vascular fraction

White adipocytes and the stromal vascular fraction (SVF) were isolated from epididymal white adipose tissue (AT) according to previously described methodology [18]. Isolated adipocyte and SVF preparations were incubated for 3.5 h at 37°C in DMEM containing 25 mmol/l glucose. The medium was then collected and analysed for eNAMPT content by ELISA (Caltag).

### Statistical analysis

Results are expressed as the mean ± SEM. Statistical differences were calculated by one-way ANOVA and the Tukey’s test where appropriate (GraphPad Software; la Jolla, CA, USA).

## Results

### High-fat feeding selectively induces the production and secretion of eNAMPT-monomer

**Fig. 1:**
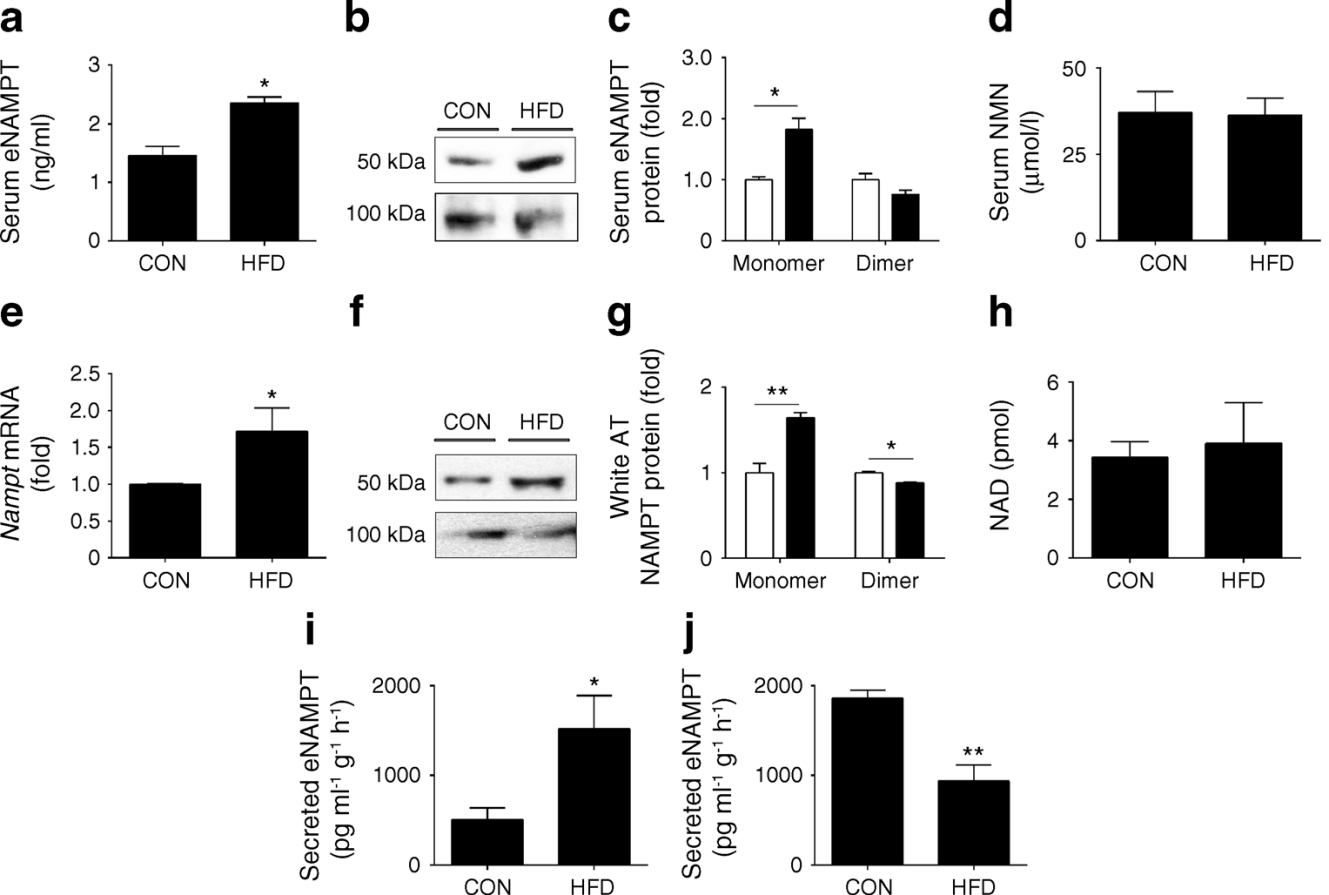
eNAMPT-monomer levels are selectively elevated in HFD-fed mice. (**a**) Total serum eNAMPT, (**b**, **c**) serum eNAMPT monomeric and dimeric protein and (**d**) serum NMN levels in CON- or HFD-fed mice (fed for 10 weeks); in (**c**) white bars, CON-fed; black bars, HFD-fed. (**e**) *Nampt* mRNA expression (normalised to control levels [levels in CON-fed mice]), (**f**, **g**) NAMPT-monomer and NAMPT-dimer protein levels (normalised to control [CON-fed]) and (**h**) total NAD levels in white AT (in (**c**) and (**g**), white bars, CON-fed; black bars, HFD-fed). eNAMPT secretion from (**i**) SVF and (**j**) white adipocytes. Western blots are representative of three separate blots. *p < 0.05, **p < 0.01

We first demonstrated that serum eNAMPT levels were elevated in diabetic HFD-fed mice (Fig. 1a).

Following this, we examined whether increased total eNAMPT was associated with specific changes in eNAMPT-monomer or eNAMPT–dimer levels using non-reducing SDS-PAGE and immunoblotting. Indicative of a selective diabetogenic function, eNAMPT-monomer protein levels were markedly elevated in serum (82.0 ± 1.9%; Fig. 1b, c) of HFD-fed mice compared with CON-fed mice. In contrast, serum levels of the eNAMPT-dimer and its reaction product, NMN, were non-significantly reduced or unchanged between HFD- vs CON-fed mice (Fig. 1b–d). Similar changes were observed in white AT, a major source of circulating eNAMPT. White AT from HFD-fed mice had increased *Nampt* mRNA levels (Fig. 1e), with parallel increases in the NAMPT-monomer (64.0 ± 5.9%), but no observed changes in NAMPT-dimer or NAD (Fig. 1f–h). Therefore, alterations in the levels and structure of NAMPT in white AT provide a potential explanation for increased serum eNAMPT-monomer and eNAMPT-dimer levels in HFD-fed mice.

To further examine the source of eNAMPT, we isolated white adipocytes and SVF from epididymal white AT and measured eNAMPT secretion. In white adipocytes from HFD-fed mice eNAMPT secretion was markedly decreased, whilst eNAMPT secretion was increased in SVF isolated from HFD-fed mice (Fig. 1i, j). Collectively, this suggests that white adipocytes and SVF are likely to be the primary source of eNAMPT-dimer and eNAMPT-monomer, respectively. The cellular source of eNAMPT-monomer within the SVF requires further study. However, previous studies have reported eNAMPT secretion from undifferentiated pre-adipocytes and immune cells, including macrophages, neutrophils and B cells.

Together these data demonstrate that serum levels of eNAMPT-monomer are selectively elevated in diabetic HFD-fed mice. This implies a specific pathophysiological role for eNAMPT-monomer in experimental diabetes.

### Anti-Anti-eNAMPT antibody improves glycaemic control and insulin resistance in HFD-fed mice

To determine the importance of raised serum eNAMPT-monomer levels in experimental diabetes, HFD- and CON-fed mice were injected i.p. with an anti-eNAMPT antibody (eNAMPT-Ab) or non-immune IgG equivalent (2 doses/week in weeks 9–10). Immunoneutralisation enables the selective inhibition of circulating eNAMPT (without inhibiting iNAMPT). Immunoprecipitation of NAMPT with this antibody followed by immunoblotting led to detection of a 50 kDa protein band but not a 100 kDa protein band (ESM Fig. 1a, b), indicating antibody specificity for the eNAMPT-monomer. Moreover, eNAMPT-Ab blocked NAD-independent eNAMPT-mediated decreases in GSIS in MIN6 cells but did not have any effect on levels of NMN (the eNAMPT-dimer reaction product) in HFD- or CON-fed mice (ESM Fig. 1c, d). This result suggests that the eNAMPT-Ab selectively targets the eNAMPT-monomer. Serum levels of total eNAMPT were non-significantly reduced following eNAMPT-Ab treatment (ESM Fig. 1e).

**Fig. 2:**
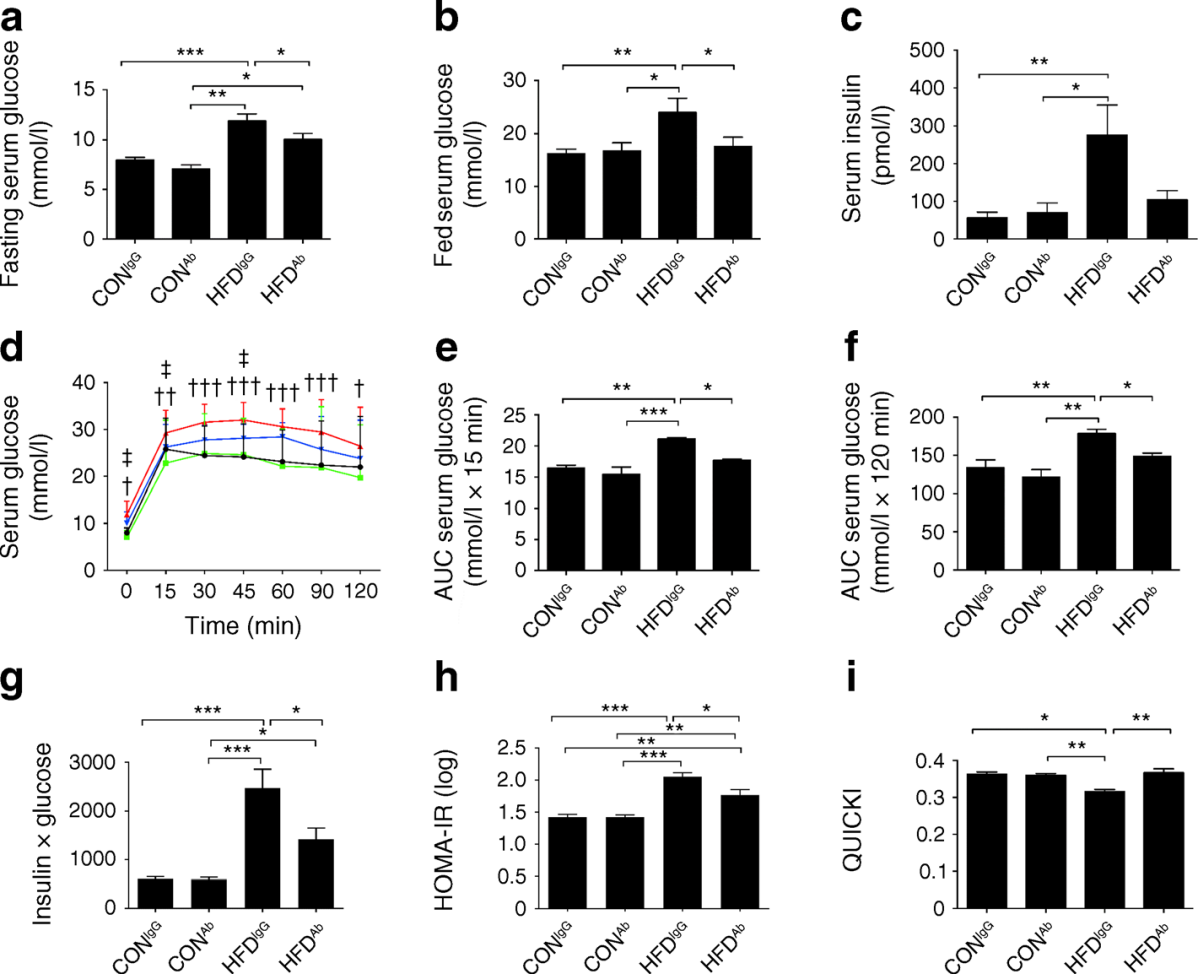
eNAMPT-monomer immunoneutralisation reverses the diabetic phenotype in HFD-fed mice. (**a**) Fasting serum glucose, (**b**) fed serum glucose, (**c**) fed serum insulin, (**d**–**f**) glucose response to IPGTT (black, CON^IgG^; green, CON-fed mice, administered eNAMPT-antibody [CON^Ab^]; red, HFD^IgG^; blue, HFD^Ab^) in CON- and HFD-fed mice (fed for 10 weeks) treated with eNAMPT-Ab or non-immune IgG. (**g**) Insulin × glucose product, (**h**) HOMA-IR and (**i**) QUICKI. *p < 0.05, **p < 0.01, ***p < 0.001 for all subparts except (**d**) where ^†^ *p* < 0.05, ^††^ *p* < 0.01, ^†††^ *p* < 0.001 for CON^IgG^ vs HFD^IgG^ and ^‡^ *p* < 0.05 for HFD^IgG^ vs HFD^Ab^

In support of a diabetogenic role for the eNAMPT-monomer, eNAMPT-Ab administration reduced blood glucose and insulin levels (Fig. 2a–c) and corrected impaired glucose tolerance (IGT) in HFD-fed mice (Fig. 2d–f). As determined by assessment of the insulin × glucose product, HOMA-IR and QUICKI, eNAMPT-Ab also ameliorated whole-body insulin resistance (Fig. 2g–i). Improved glucose tolerance and insulin sensitivity were associated with reduced fasting serum triacylglycerol and fed serum NEFA levels, although the latter did not reach statistical significance. Fed serum triacylglycerol levels were unchanged across all groups (ESM Fig. 2a–c). Body weight and food intake were also unchanged (ESM Fig. 2d, e).

Together, these data demonstrate that neutralising the eNAMPT-monomer leads to a marked improvement in the diabetic phenotype of HFD-fed mice.

### eNAMPT-Ab improves pancreatic islet function and increases islet size in HFD-fed mice

Type 2 diabetes is characterised by progressive pancreatic beta cell failure [1]. Therefore, we assessed whether eNAMPT-Ab mediated improvements in glycaemic control via improvements in beta cell/islet health.

**Fig. 3:**
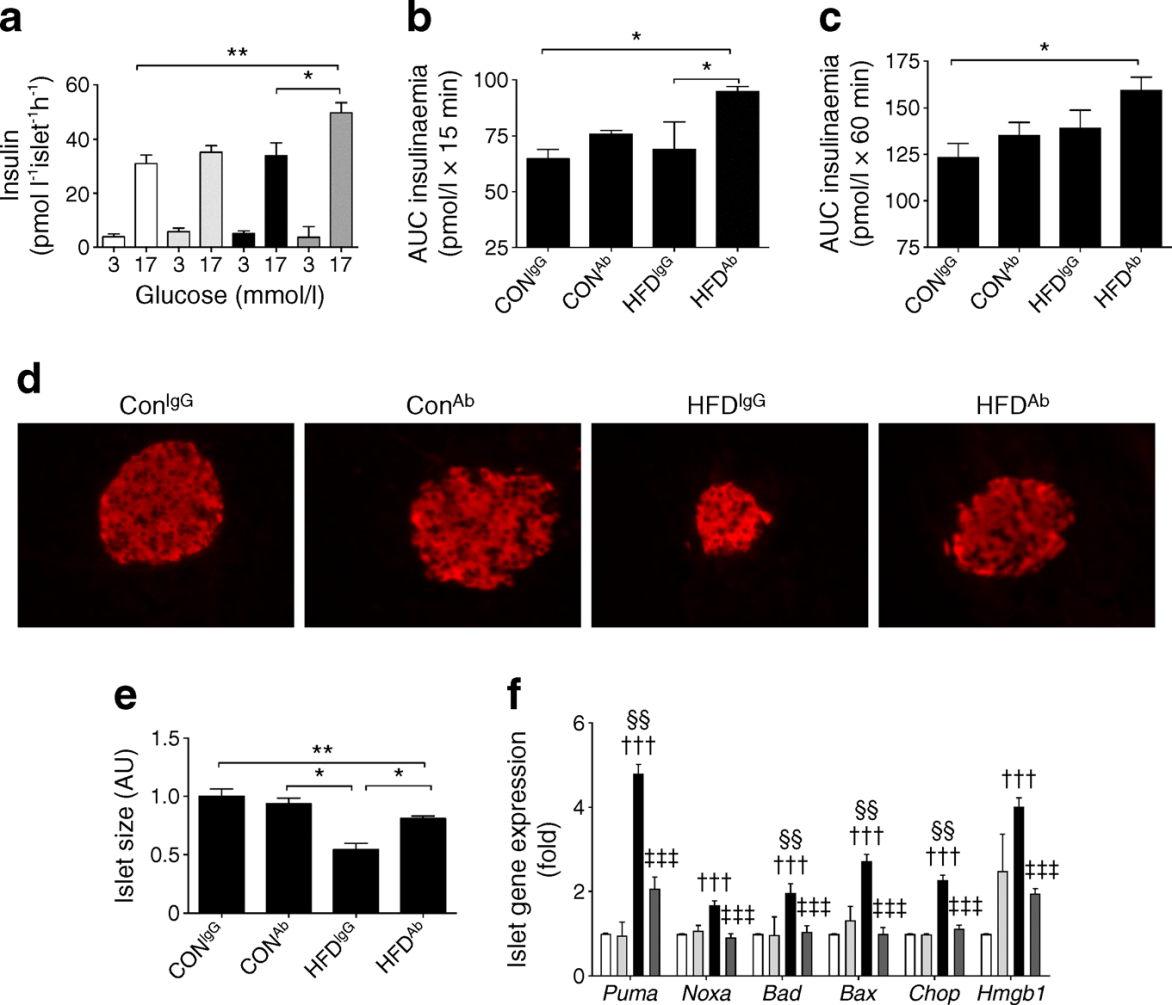
eNAMPT immunoneutralisation reverses beta cell dysfunction in HFD-fed mice. (**a**) Ex vivo GSIS and (**b**, **c**) AUC insulin response to IPGTT in CON- and HFD-fed mice (fed for 10 weeks) i.p. injected with eNAMPT-Ab or non-immune IgG. (**d**) Insulin immunofluorescence staining of whole pancreatic sections (magnification ×20), (**e**) relative islet size (AU, arbitrary unit) and (**f**) gene expression of pro-apoptotic genes in isolated islets (normalised to control [CON^IgG^]). (**a**, **f**) white, CON^IgG^; light grey, CON^Ab^; black, HFD^IgG^; dark grey, HFD^Ab^. *p < 0.05, ** *p* < 0.01 for all subparts except (**f**) where ^†††^ *p* < 0.001 for CON^IgG^ vs HFD^IgG^, ^‡‡‡^ *p* < 0.001 for HFD^IgG^ vs HFD^Ab^ and ^§§^ *p* < 0.01 for CON^Ab^ vs HFD^IgG^

Beta cell/islet function was impaired in HFD-fed mice, as evidenced by the lack of an islet compensatory response to insulin resistance in static ex vivo GSIS studies (Fig. 3a), and in vivo measurements of the first (0–15 min) and second phase (15–60 min) insulin secretory responses to glucose (Fig. 3b, c). Crucially, eNAMPT immunoneutralisation restored islet compensation, as demonstrated by a marked increase in ex vivo GSIS and in vivo first phase insulin secretion in HFD-fed mice, administered eNAMPT-antibody (HFD^Ab^).

We next assessed the effects of the eNAMPT-Ab on islet size. Islet size was reduced by 46% (*p <* 0.01) in HFD-fed mice, administered IgG (HFD^IgG^) compared with CON-fed mice, administered IgG (CON^IgG^); this effect was completely reversed by eNAMPT-Ab treatment (Fig. 3d, e). Several studies have observed an increased islet size in HFD-fed mice. However, in this study the reduced islet size observed is likely to reflect progression from insulin resistance to an overt diabetic phenotype in our model. Beta cell apoptosis and necrosis are commonly considered to be mechanisms responsible for reduced beta cell/islet mass in type 2 diabetes. Consistent with this, islet mRNA levels of pro-apoptotic and necrotic markers were significantly elevated in HFD-fed mice (Fig. 3f). Strikingly, mRNA levels of these genes were lowered to basal levels following eNAMPT-Ab administration, demonstrating that eNAMPT immunoneutralisation improves beta cell function/mass in part by protecting against beta cell apoptosis and necrosis. Together, these data demonstrate that eNAMPT immuno-neutralisation improves glycaemic control in HFD-fed mice in part through reversing beta cell dysfunction and restoring islet compensation.

### eNAMPT-Ab improves hepatic insulin sensitivity and reduces hepatic fat content

Obesity-induced non-alcoholic fatty liver disease (NAFLD), characterised by excess hepatic lipid accumulation and inflammation, is an established risk factor for the development of hepatic insulin resistance and type 2 diabetes [19]. Elevated serum eNAMPT levels in NAFLD are associated with worsening disease severity [20]. Consistent with a potential role for eNAMPT-monomer in the development of fatty liver, the eNAMPT-Ab reversed HFD-mediated increases in hepatic triacylglycerol content (ESM Fig. 3a) and changes in *Srebf1* and *Fasn* lipogenic gene expression (ESM Fig. 3b). Hepatic insulin resistance is strongly associated with increased liver lipid content. Moreover, eNAMPT-Ab reversed HFD-mediated decreases in hepatic protein levels of -p-Akt (Ser^473^), a marker of insulin signalling (ESM Fig. 3c), and corrected abnormally elevated mRNA levels of *Pck1* (ESM Fig. 3b), a key gluconeogenic gene. Together, these data suggest that eNAMPT immunoneutralisation lowers hepatic lipid content and may improve hepatic insulin sensitivity in HFD-fed mice.

### eNAMPT-Ab ameliorates tissue and systemic inflammation in HFD-fed mice

**Fig. 4:**
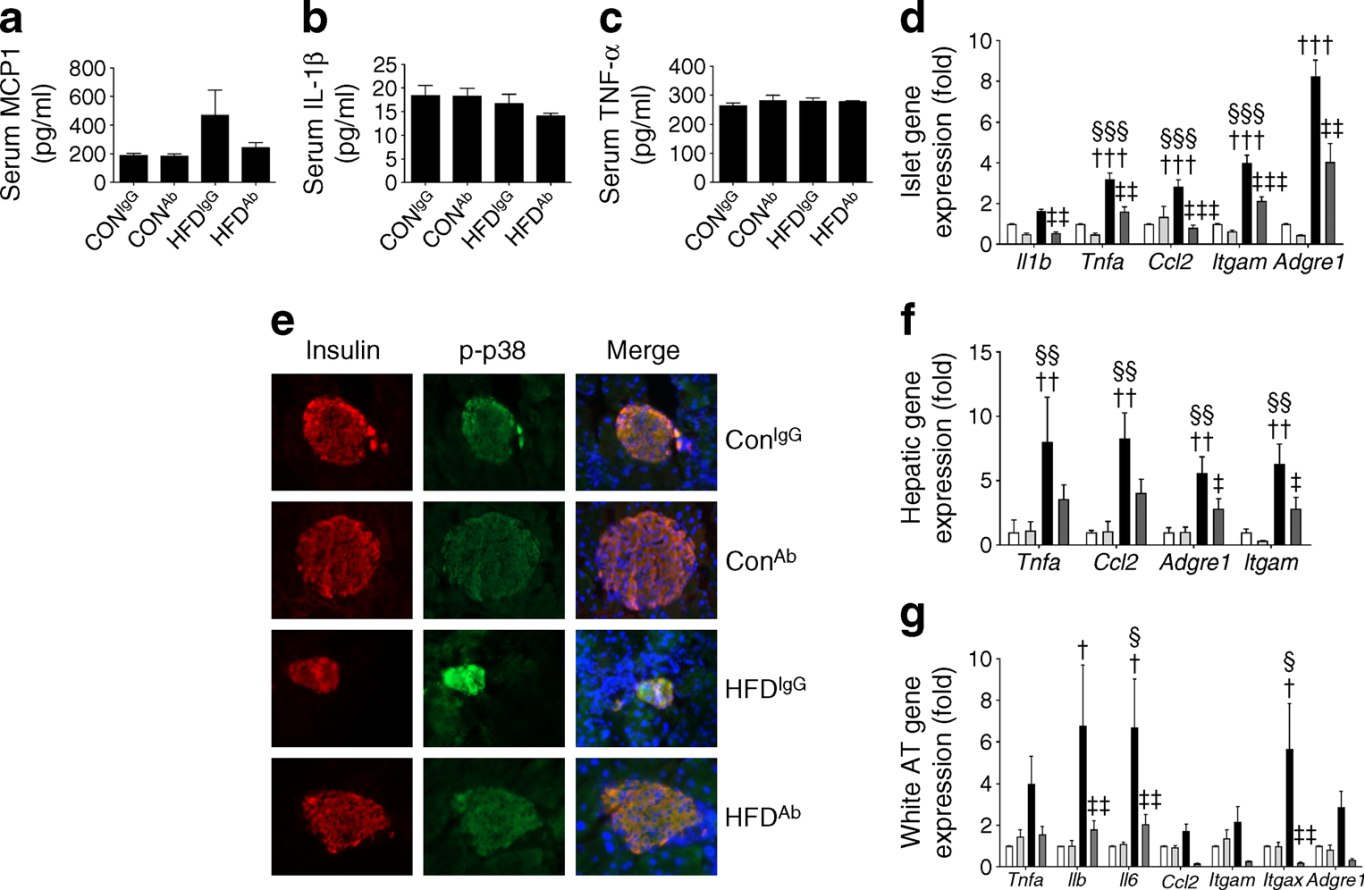
eNAMPT-monomer immunoneutralisation improves tissue and systemic inflammation in HFD-fed mice. Serum concentrations of (**a**) MCP1, (**b**) IL-1β and (**c**) TNF-α in CON- and HFD-fed mice (fed for 10 weeks) injected i.p. with eNAMPT-Ab or non-immune IgG. (**d**) Gene expression of proinflammatory genes in isolated islets, (**e**) insulin and p-p38 islet immunofluorescence staining of whole pancreatic sections (magnification ×20), (**f**) hepatic expression of proinflammatory genes and (**g**) white AT proinflammatory gene expression (normalised to control [CON^IgG^]). (**e**, **f** ) white, CON^IgG^; light grey, CON^Ab^; black, HFD^IgG^; dark grey, HFD^Ab^. ^†^ *p* < 0.05, ^††^ *p* < 0.01 and ^†††^ *p* < 0.001 for CON^IgG^ vs HFD^IgG^; ^‡^ *p* < 0.05, ^‡‡^ *p* < 0.01 and ^‡‡‡^ *p* < 0.001 for HFD^IgG^ vs HFD^Ab^; and ^§^ *p* < 0.05, ^§§^ *p* < 0.01 and ^§§§^ *p* < 0.001 for CON^Ab^ vs HFD^IgG^

Chronic inflammation causes beta cell failure in type 2 diabetes [21]. Proinflammatory functions of eNAMPT have been reported, although studies have not distinguished between monomeric and dimeric forms [10], nor have they examined a specific proinflammatory role for eNAMPT in type 2 diabetes. The eNAMPT-Ab lowered HFD-mediated increases in serum monocyte chemoattractant protein (MCP-1; also known as chemokine (C-C motif) ligand 2[CCL2]) levels (Fig. 4a), although serum TNF-α and IL-1β levels were similar in all experimental groups (Fig. 4b, c). Furthermore, eNAMPT-Ab reversed HFD-mediated increases in islet mRNA levels of proinflammatory cytokines (*Tnfa*, *Il1b*, *Il6*), chemokines (*Ccl2*, encoding MCP1) and immune cell markers (*Itgam*, encoding CD11b; *Itgax*, encoding CD11c; *Adgre1*, encoding F4/80; Fig. 4d). Studies in monocytes have reported that eNAMPT induces proinflammatory cytokine production in part through activation of p38 mitogen-activated protein kinase (MAPK) [10]. In agreement, we found that the islet p-p38 immunofluorescence signal (denoting activated p38) was enhanced in HFD-fed mice compared with CON-fed mice, and that this effect was reversed by eNAMPT-Ab treatment (Fig. 4e). Chronic inflammation in liver and white AT is also common in obesity and type 2 diabetes, where it plays a crucial role in disease pathophysiology. Consistent with a proinflammatory effect for the eNAMPT-monomer in these tissues, eNAMPT-Ab lowered the expression of proinflammatory genes in the liver and AT of HFD-fed mice (Fig. 4f, g). These data provide strong support for the notion that eNAMPT immunoneutralisation improves pancreatic beta cell health and peripheral insulin resistance through resolution of inflammation in HFD-fed mice, and that the eNAMPT-monomer is likely to exert proinflammatory effects.

### The beneficial effects of eNAMPT-Ab are maintained 3 weeks post-treatment

**Fig. 5:**
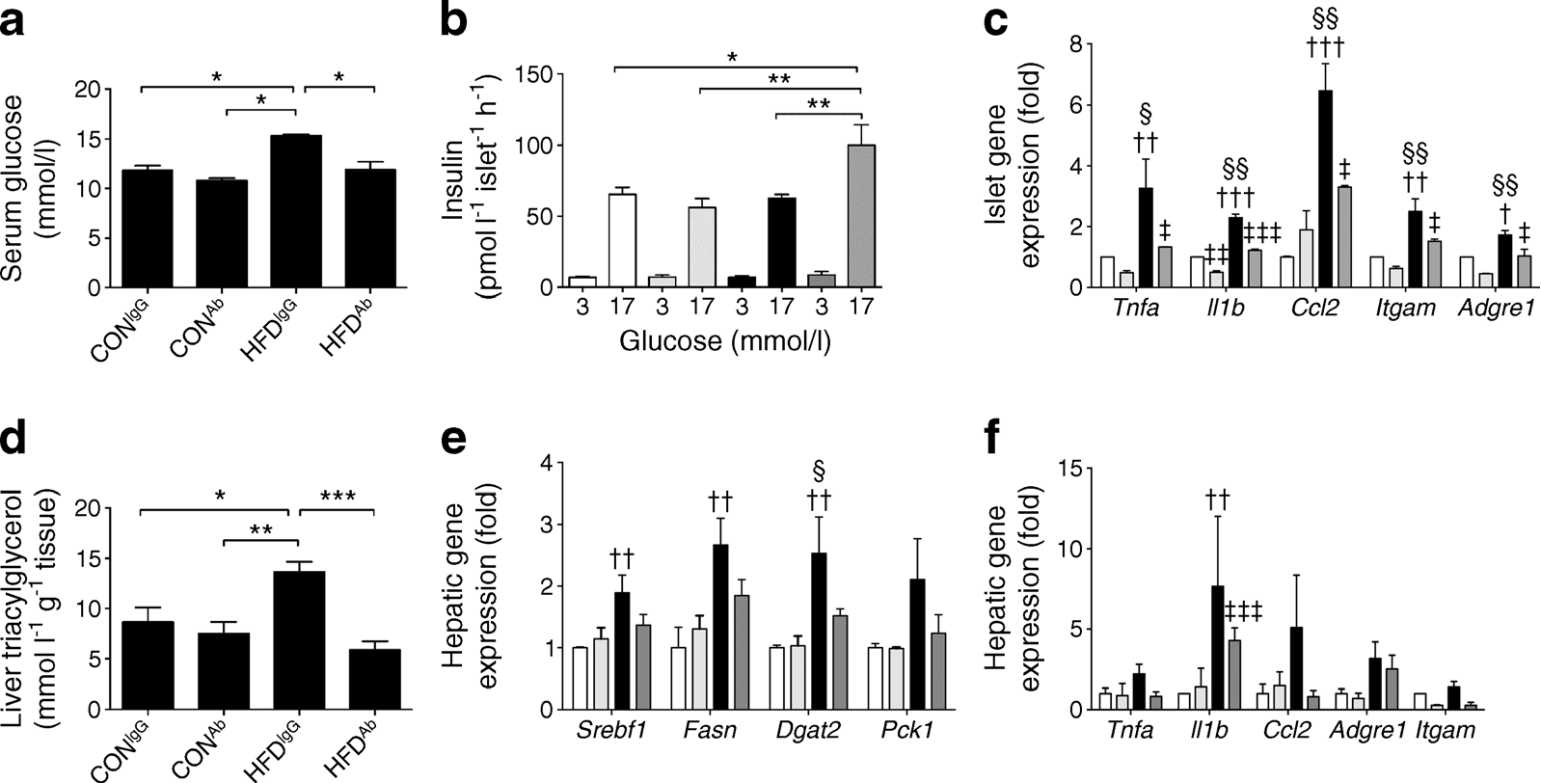
The beneficial effects of eNAMPT-monomer immunoneutralisation in HFD-fed mice are maintained for 3 weeks post-treatment. (**a**) Fed serum glucose levels, (**b**) ex vivo GSIS, (**c**) islet expression of proinflammatory genes, (**d**) hepatic triacylglycerol levels, (**e**) hepatic expression of lipogenic and gluconeogenic genes and (**f**) hepatic expression of proinflammatory genes in CON- and HFD-fed mice (fed for 13 weeks), injected i.p. with eNAMPT-Ab or non-immune IgG (at weeks 9–10; **c**, **e**, **f**: normalised to control [CON^IgG^]). In (**b**, **c**, **e**, **f**), white, CON^IgG^; light grey, CON^Ab^; black, HFD^IgG^; dark grey, HFD^Ab^. *p < 0.05; **p < 0.01, ***p < 0.001 for all subparts except (**c**, **e**, **f**) where ^†^ *p* < 0.05, ^††^ *p* < 0.01 and ^†††^ *p* < 0.001 for CON^IgG^ vs HFD^IgG^, ^‡^ *p* < 0.05, ^‡‡^ *p* < 0.01, ^‡‡‡^ *p* < 0.001 for HFD^IgG^ vs HFD^Ab^ and ^§^ *p* < 0.05 and ^§§^ *p* < 0.01 for CON^Ab^ vs HFD^IgG^

To determine whether the effects of eNAMPT immunoneutralisation were maintained over time, eNAMPT-Ab was administered in weeks 9–10, as previously described. Mice then remained on CON or HFD diets until week 13 without further eNAMPT-Ab administration. At 3 weeks post-treatment, the eNAMPT-Ab-lowered HFD-mediated increases in blood glucose enhanced islet insulin secretion, reduced islet inflammation, and reduced hepatic triacylglycerol content, hepatic lipogenic gene expression and hepatic inflammation (Fig. 5). Thus, the beneficial effects of a single dose regimen of eNAMPT-Ab were maintained for at least 3 weeks post treatment.

### eNAMPT-monomer administration for 14 days induces a diabetic phenotype in mice

**Fig. 6:**
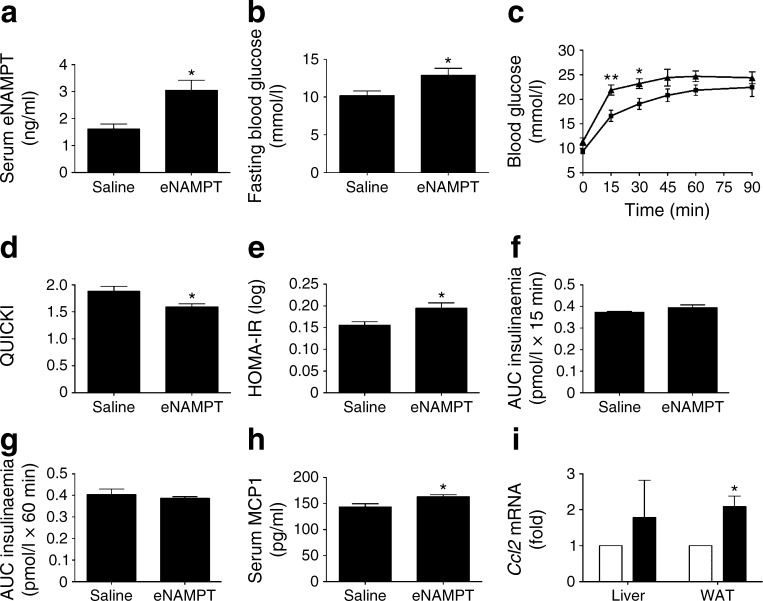
eNAMPT-monomer administration for 14 days induces a diabetic phenotype in mice. (**a**) Serum eNAMPT levels, (**b**) blood glucose levels, (**c**) IPGTT (squares, saline; triangles, eNAMPT), (**d**) QUICKI and (**e**) HOMA-IR in mice injected i.p. with recombinant eNAMPT-monomer or the equivalent volume of 154 mmol/l NaCl. AUC insulin response to IPGTT at (**f**) 0–15 min and (**g**) 15–60 min, (**h**) serum MCP1 levels and (**i**) *Ccl2* gene expression (white bars, saline; black bars, eNAMPT). *p < 0.05, **p < 0.01

Finally, we examined the impact of experimental elevation of serum eNAMPT-monomer levels on glycaemic control in mice. Lean, non-diabetic mice were injected i.p. with recombinant eNAMPT or the equivalent volume of NaCl (154 mmol/l). eNAMPT administration doubled serum eNAMPT levels to 3.05 ± 0.37 ng/ml (Fig. 6a), similar to levels in HFD-fed mice. When recombinant eNAMPT protein was analysed by non-reducing SDS-PAGE and immunoblotting, a 50 kDa protein band was detected but a 100 kDa protein band was not (ESM Fig. 1f). Moreover, recombinant eNAMPT treatment did not increase NAD levels in vitro in MIN6 cells, nor in vivo in mouse white AT (ESM Fig. 1g, h). These findings suggest that the recombinant eNAMPT used in this study does not dimerise to promote NAD biosynthesis, and thus represents monomeric eNAMPT. Therefore, to mimic elevated dimeric eNAMPT, a separate group of mice were injected i.p. with NMN, the reaction product of the eNAMPT-dimer’s NAD biosynthetic reaction.

The 14-day eNAMPT-monomer administration resulted in elevated fasting blood glucose levels (Fig. 6b), development of IGT (0–30 min post-glucose injection; Fig 6c) and whole-body insulin resistance, as determined by QUICKI and HOMA-IR (Fig. 6d, e). These changes were not related to changes in body weight, serum triacylglycerol or insulin levels (ESM Fig. 4a–d). Moreover, eNAMPT-monomer administration led to impaired insulin secretion, as demonstrated by a lack of islet compensatory response to insulin resistance (Fig. 6f, g). Consistent with its hypothesised proinflammatory actions, eNAMPT-monomer administration led to elevated serum of MCP1 levels (Fig. 6h) and increased liver and white AT *Ccl2* mRNA levels (Fig. 6i), although serum IL-1β and TNF-α levels and liver and white AT mRNA levels of a number of other proinflammatory markers remained unchanged (ESM Fig. 4e–h). eNAMPT-monomer administration induced a non-significant trend towards increased proinflammatory gene expression in islets (ESM Fig. 4i).

In contrast to the diabetogenic effects of the eNAMPT-monomer, NMN administration resulted in mild reductions in blood glucose and serum IL-1β levels, without changes in body weight, glucose tolerance or insulin sensitivity (ESM Fig. 5).

Together, these data suggest distinct structure–function characteristics of eNAMPT monomers and dimers; the eNAMPT-monomer induced a diabetic phenotype in mice via NAD-independent proinflammatory effects, whilst eNAMPT-dimer/NMN elevation led to mild improvements in glycaemic control.

## Discussion

We provide multiple lines of evidence suggesting an important NAD-independent role for the eNAMPT-monomer in type 2 diabetes: (1) diabetic HFD-fed mice displayed a selective increase in serum eNAMPT-monomer levels; (2) blocking eNAMPT action reversed the diabetogenic effects of HFD; and (3) 14-day administration of the eNAMPT-monomer induced a diabetic phenotype in non-diabetic mice.

Elevated serum eNAMPT levels are reported in type 2 diabetes [2, 3, 20, 22–24], although other studies have observed decreased or unchanged serum eNAMPT levels in metabolic diseases [25, 26].

Our findings go some way in clarifying the contradictory findings regarding eNAMPT and type 2 diabetes by demonstrating differences in the levels of eNAMPT-monomer and eNAMPT-dimer in experimental diabetes and highlighting the relevance of structure–function differences between monomer and dimer.

The precise function of eNAMPT and the relationship between raised eNAMPT levels and type 2 diabetes pathophysiology remain unresolved. Previous experimental studies have demonstrated both beneficial and deleterious effects of recombinant eNAMPT on insulin secretion and sensitivity in vitro [27–30]. However, these studies have often examined the acute effects of eNAMPT, which are unlikely to accurately represent a diabetic phenotype, or have used supraphysiological concentrations of eNAMPT. Crucially, these studies also did not distinguish between eNAMPT-monomers and eNAMPT-dimers.

Our studies, and those of other groups, have described beneficial acute and chronic effects of NMN, the reaction product of the eNAMPT-dimer biosynthetic reaction [4, 6, 7, 31, 32]. Consistent with a beneficial effect of the eNAMPT-dimer, serum and white AT eNAMPT-dimer levels were unchanged or decreased in HFD-fed mice, whilst 14-day NMN administration lowered blood glucose and inflammation in non-diabetic mice. In contrast, we describe a specific role for the eNAMPT-monomer in experimental diabetes via functioning partly through NAD-independent proinflammatory effects. Proinflammatory effects of eNAMPT in type 2 diabetes have not previously been reported. However, our findings are broadly in agreement with studies of eNAMPT in other diseases, although such studies did not distinguish between monomers and dimers. For example, eNAMPT is reported to induce monocyte expression and secretion of IL-6, IL-1β and TNF-α [10], potentially via receptor-mediated mechanisms [33]. Thus, normalisation of eNAMPT-monomer signalling in type 2 diabetes would be predicted to resolve inflammation-mediated beta cell dysfunction and insulin resistance and improve glycaemic control.

We hypothesise that increased monomer levels are partly related to AT dysfunction. AT-derived eNAMPT-dimer is secreted from fully differentiated adipocytes, whilst the monomer may be secreted from poorly-differentiated adipocytes and immune cells [5, 9, 10, 34–36]. Since poor adipocyte differentiation and immune cell infiltration are characteristics of obese AT [37], we hypothesise that such pathophysiological changes could explain the increased monomer levels in HFD-fed mice. In agreement, eNAMPT secretion from the AT SVF (which contains immune cells and undifferentiated pre-adipocytes) was markedly increased in HFD-fed mice. Thus, we hypothesise that obesity-mediated AT dysfunction results in a phenotypic switch, characterised by elevated eNAMPT-monomer production from SVF and reduced dimer secretion from adipocytes. Future studies will elucidate the precise mechanisms involved in dimer formation and identify the main cellular source of eNAMPT within the SVF.

Together, these studies suggest that the eNAMPT-monomer is an attractive therapeutic target for type 2 diabetes. Future strategies to develop this therapeutic approach will include development of eNAMPT-monomer receptor antagonists, specific eNAMPT-monomer inhibitors and humanised eNAMPT monoclonal antibodies. Humanised monoclonal antibodies can be engineered with an extended *t*½, potentially allowing clinical benefits to be achieved with only one dose every 1–3 months [38], thus providing benefits in terms of cost, convenience and compliance.

In summary, we have demonstrated that elevated eNAMPT-monomer levels contribute to the development of type 2 diabetes. In addition, we have provided proof-of-concept evidence that selectively blocking the action of eNAMPT-monomer is a promising therapeutic strategy for the treatment of type 2 diabetes.

## Electronic supplementary material

Below is the link to the electronic supplementary material.

ESM (PDF 803 kb)

## Abbreviations

AT: Adipose tissue
CCL2: Chemokine (C-C motif) ligand 2
CON: Control diet
CON^Ab^: Control diet-fed mice, administered eNAMPT-antibody
CON^IgG^: Control diet-fed mice, administered IgG
HFD: High-fat diet
HFD^Ab^: High-fat diet-fed mice, administered eNAMPT-antibody
HFD^IgG^: High-fat diet-fed mice, administered IgG
IGT: Impaired glucose tolerance
iNAMPT: Intracellular nicotinamide phosphoribosyltransferase
eNAMPT: Extracellular nicotinamide phosphoribosyltransferase
GSIS: Glucose-stimulated insulin secretion
HFD: High-fat diet
iNAMPT: Intracellular nicotinamide phosphoribosyltransferase
MCP1: Monocyte chemoattractant protein
NAFLD: Non-alcoholic fatty liver disease
NAMPT: Nicotinamide phosphoribosyltransferase
NMN: Nicotinamide mononucleotide
PBEF: Pre-B cell colony-enhancing factor
qRT-PCR: Quantitative RT-PCR
SVF: Stromal vascular fraction
