# Supplementary material for: Monomeric eNAMPT in the development of experimental diabetes in mice: a potential target for type 2 diabetes treatment
Source: Diabetologia. 2016 Aug 19;59(11):2477–86. doi: 10.1007/s00125-016-4076-3 (PMC5506101; doi:10.1007/s00125-016-4076-3)
Supplement: Supplementary file 1 — (PDF 803 kb) [file 125_2016_4076_MOESM1_ESM.pdf]

ESM Materials and Methods

Serum NMN measurements

NMN was detected fluorometrically in serum using high-performance liquid chromatography (HPLC), using a modified version of a previously described methodology (37). Plasma samples (30 µl) were extracted with 100 µl perchloric acid (1 mol/L) and then neutralized by addition of 330 µl K_2CO_3 (3 mol/L) followed by incubation at 4ºC for 10 min. Serum samples and standard solutions of NMN (30 µl; 25 – 200 µM) were subsequently derivatised by addition of 100 µl KOH (1 mol/L) and 50 µl acetophenone (Sigma, Poole, UK) followed by incubation at 4ºC for 15 min. Formic acid (100 µl) was then added and the solution incubated for 5 min at 100ºC, producing a highly fluorescent compound. Samples or standards were injected into the HPLC system consisting in a mobile phase of 0.1 M ammonium acetate and 1 mM EDTA buffer pH 5.65, 15% acetonitrile, a C18 column (15 cm length; 2 mm internal diameter) and a fluorometric detector (FP-920 Intelligent Fluorescence Detector; JASCO, Essex, UK) with excitation and emission wavelength of 332 and 454 nm, respectively.

Islet isolation

Islet isolation was conducted as previously described (6). Mouse pancreases were digested in 2 ml Hanks Buffered Salt Solution (HBSS) containing collagenase P (1 mg/ml) and DNAse I (0.15 mg/ml; both Roche Diagnostics, Burgess Hill, UK). Islets were hand-picked into RPMI 1640 media (containing 11 mol/L glucose, supplemented with 10% (v/v) heat-inactivated FBS; 100 U/ml penicillin and 100 µg/ml streptomycin; all Sigma Aldrich, Poole, UK). Isolated islets were either picked into RPMI and immediately lysed for RNA extraction or transferred to RPMI and allowed to recover for 2 h, prior to *ex vivo* insulin secretion assay.

Insulin Secretion *ex vivo*

Islet insulin-secretion assays were conducted as previously described (6). Briefly, batches of ten size-matched islets were pre-incubated for 1 h at 37ºC in HBSS containing 3 mM glucose, 10 mM HEPES (pH 7.4) and 0.2% BSA (w/v). For glucose-stimulated insulin secretion, islets were incubated for 1 h at 37ºC in HBSS (10 mol/L HEPES (pH 7.4), 0.2% w/v BSA) supplemented with 3 mol/L or 17 mol/L glucose. After 1 h media was collected and insulin levels were determined using a specific ELISA (Mercodia, Uppsala, Sweden)

Immunofluorescence of Mouse Pancreatic Sections

Islet immunostaining (27) for insulin and phospho-p38 was performed on pancreas sections that had been fixed in buffered paraformaldehyde (3.8%) and paraffin-embedded. Sections were incubated overnight at 4˚C in guinea-pig anti-insulin antibody (1:100; Abcam, Cambridge, UK) and/or rabbit anti-phospho p38 (Thr^{180}/Tyr^{182}; Cell Signaling Technologies, MA, USA) antibody (1:1600) and detected with goat anti-guinea pig AlexaFluor® 647 (1:1000) and goat anti-rabbit AlexaFluor® 488 (1:1000) conjugated secondary antibody (Invitrogen, Waltham, MA, USA), respectively. DAPI (1:1000, Invitrogen, Waltham, MA, USA) was included in the final incubation step to stain cell nuclei. Sections were mounted in Vectashield hard-set mounting medium (Vector Laboratories, Peterborough, UK) under glass cover slips. Mouse pancreatic sections were analyzed using a Leica DM5000 Epi-Fluorescent microscope and Leica Application Suite software.

MIN6 Cell culture and treatment

MIN6 beta-cells were cultured in DMEM media containing GlutaMAX, 25 mol/L glucose and Sodium pyruvate supplemented with 15% v/v foetal bovine serum, 1% v/v Penicillin/Streptomycin/Glutamine (all Life Technologies, Paisley, UK) and 5 µl β-mercaptoethanol. Cells were incubated for 48 h with recombinant eNAMPT (2 – 10 ng/ml; Adipogen, Seoul, South Korea) with or without eNAMPT-Ab (2.5 µg/ml). After 48 h treatments cells were analysed for changes in glucose-stimulated insulin secretion or NAD levels.

ESM Table

Tnfa	CGGAGTCCGGGCAGGT	GCTGGGTAGAGAATGGATGAACA
Il1b	GGGCTGCTTCCAAACCTTTG	TGATACTGCCTGCCTGAAGCTC
Il6	GGTGACAACCACGGCCTTCCC	ACAGGTCTGGGCTGCTGGTC
Adgre1	AGCACGTCCTATTICAACGGT	TCTGGAACACCACAAGAAAGTG
Ccl2	GGCTGGAGAGCTACAAGAGG	GGTCAGCACAGACCTCTCTG
Itgam	TGGACGCTGATGGCAATACC	GAGGCAAGGGACACACTGAC
Itgax	TGAGCTGTACCTGGATAGCCT	TGTGTCAGCTTCTCTGCATCC
Nampt	GCGAGCGAGCGGTGACT	CTGCGAGCAAGGAGAAAAATG
Puma	TTCATGGGACTCCTCCCCTC	GGTGTAGGCACCTAGTTGGG
Noxa	ACTGAACGGATGTTGCCTGT	CCCGGGGAAAAGATCACAGT
Bad	CTTGAGGAAGTCCGATCCCG	CATACTCTGGGCTGCTGGTC
Bax	GCTGGACACTGGACTTCCTC	GAGGCCTTCCCAGCCAC
Chop	CCTAGCTTGGCTGACAGAGG	GGGCACTGACCACTCTGTTT
Hmgb1	TTGCTTTGCCCATTTTGGGT	GGCATGTGGACAAAAGCTCTC
Srebf1	GCAGACCCTGGTGAGTGG	GTCGGTGGATGGGCAGTTT
Fasn	CACTGCATTGACGGCCGGGT	GGACAAGCCCAGGCTGCGAG
Dgat2	TCTCAGCCCTCCAAGACATC	GCCAGCCAGGTCAAGTAGAG
Pck1	TCCTGCAGAACACAAGGGC	GGTCGCATGGCAAAGGG

ESM Table 1. List of qRT-PCR primer sequences used in this study

ESM Figures and Legends

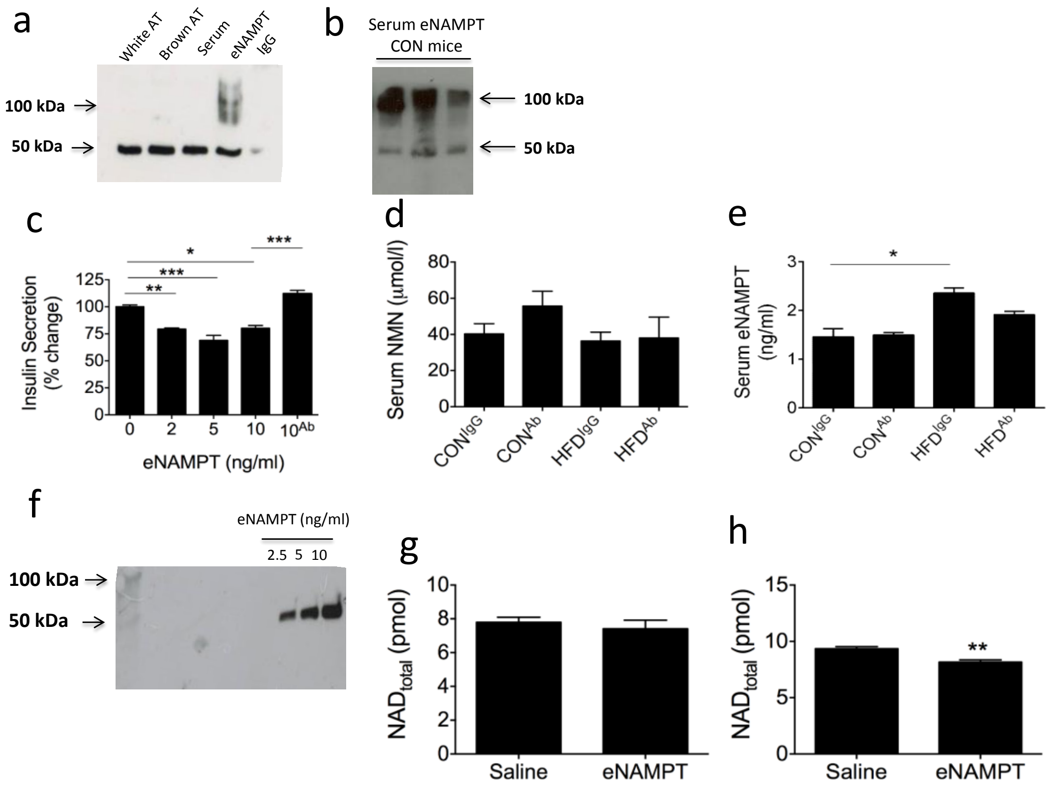

ESM Fig. 1. Specificity of eNAMPT-Ab and recombinant eNAMPT protein. (a) NAMPT/eNAMPT immunoprecipitation (with LSBio anti-NAMPT-Ab) and subsequent immunoblotting (with Bethyl Laboratories anti-NAMPT antibody); (b) serum eNAMPT monomer and dimer protein in CON fed mice, measured non-reducing SDS-PAGE and immunoblot (c) Glucose-stimulated insulin secretion from MIN6 cells following incubation with eNAMPT-monomer or co-incubated with eNAMPT-monomer and LSBio eNAMPT-Ab (48 h). Serum NMN (d) and total-eNAMPT (e) levels in 10 week-fed CON and HFD mice administered eNAMPT-Ab or control IgG. (f) Non-reducing SDS-PAGE gel and western blot of recombinant eNAMPT (Adipogen, Seoul, South Korea). NAD levels in (g) MIN6 cells following recombinant eNAMPT exposure (5 ng/ml; 48 h) and in (h) white AT following recombinant eNAMPT administration to mice (5 ng/ml/day; I.P. 14 days). Data are expressed as mean ± SEM. Statistically significant differences between groups are indicated by * $p<0.05$; ** $p<0.01$; *** $p<0.001$.

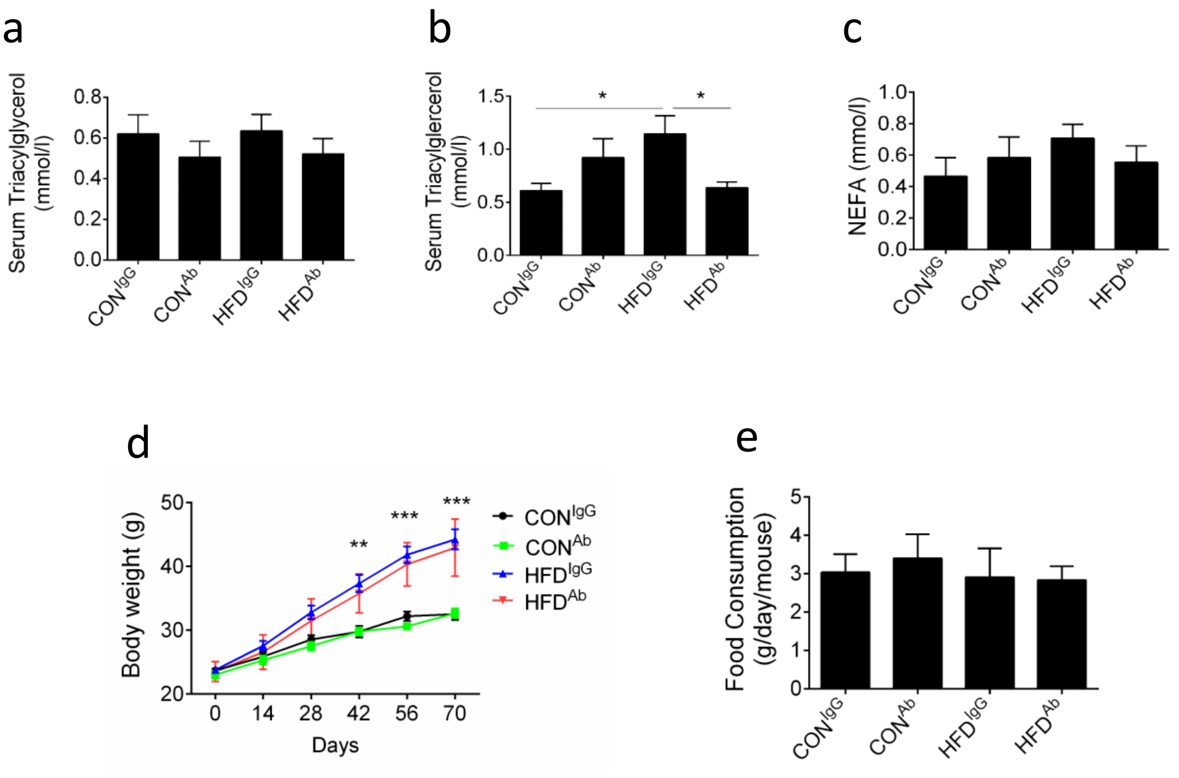

ESM Fig. 2. Effects of eNAMPT-Ab on body weight and serum lipids. CON and HFD mice (10 weeks) were administered eNAMPT-Ab or non-immune IgG; (a) Fasting serum triglyceride, (b) Fed serum triglyceride, (c) Fed serum NEFA levels, (d) Body weight, (e) Food intake post-antibody administration. Data are expressed as mean ± SEM. Statistically significant differences between are indicated by ** p<0.05; ** p<0.01; *** p<0.001;

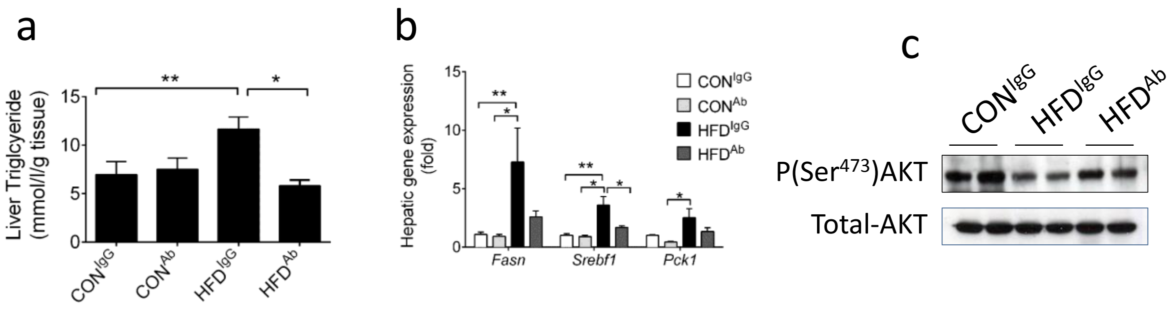

ESM Fig. 3. Effects of eNAMPT-Ab on liver insulin sensitivity and lipid levels. CON and HFD mice (10 weeks) were administered eNAMPT-Ab or non-immune IgG; (a) Liver triglycerides, (b) liver gluconeogenic and lipogenic gene expression, (c) liver phospho-AKT levels. Data are expressed as mean ± SEM. Statistically significant differences between groups are indicated by * p<0.05, ** p <0.01.

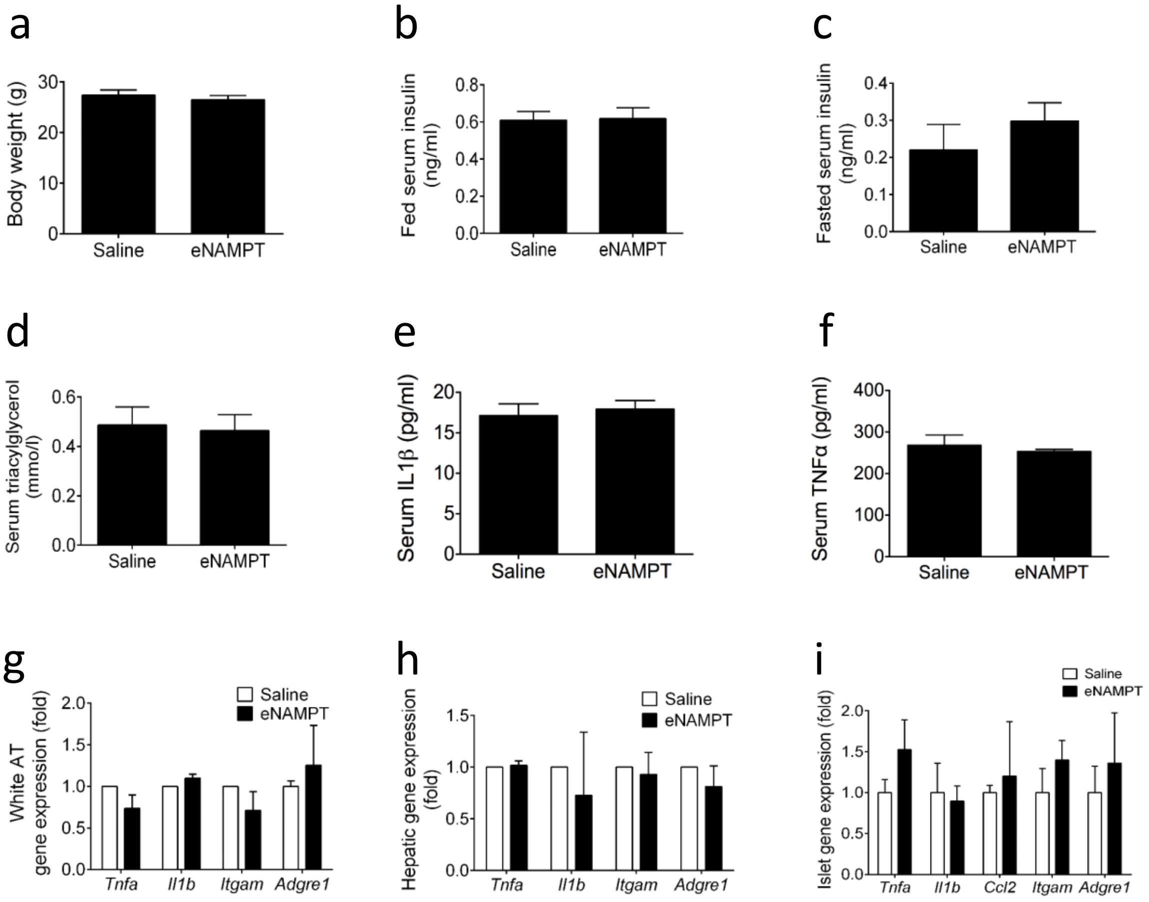

ESM Fig. 4. Effects of 14-day eNAMPT administration in mice. Mice were administered eNAMPT (5ng/ml) daily for 14 days; (a) body weight, (b) Fed serum insulin, (c) Fasting serum insulin (d) Serum Triglycerides, (e) Serum IL1β, (f) Serum TNFα. Pro-inflammatory gene expression in (g) WAT, (h) Liver, and (i) Islets. Data are expressed as mean ± SEM.

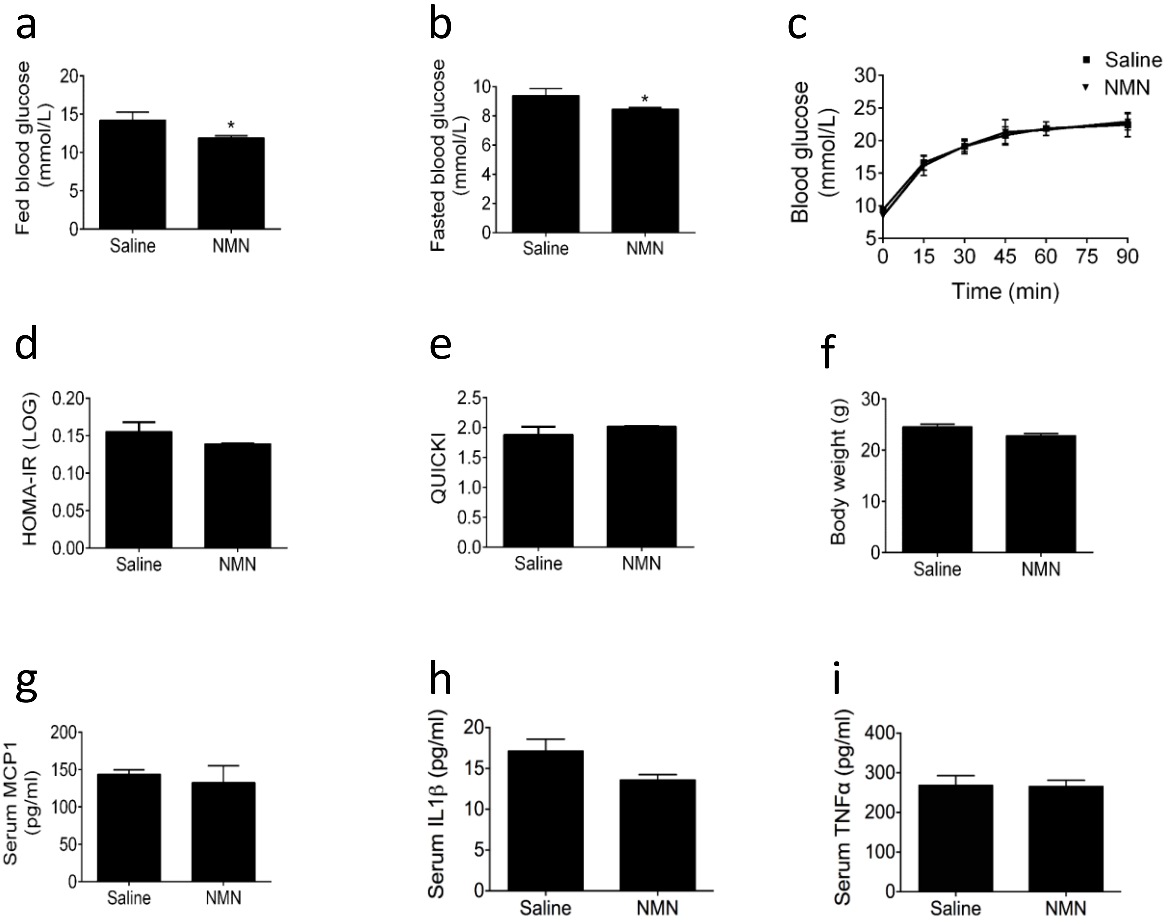

ESM Fig. 5. Effects of 14-day NMN administration in mice. Mice were administered NMN (500 mg/kg body weight) daily for 14 days; (a) Fed Blood Glucose, (b) Fasted blood glucose, (c) IPGTT; (d) HOMA-IR (LOG), (e) QUICKI; (f) Body weight. Serum levels of (g) MCP1, (h) IL1β and (i) TNFα. Data are expressed as mean ± SEM. Statistically significant differences between groups are indicated by * p<0.05.
